# Perceptions of Healthcare Professionals on Clinical Debriefing Structured by The Talk Method

**DOI:** 10.1111/jep.70641

**Published:** 2026-10-04

**Authors:** Jéssica Santos Oliveira Targino, Antônio Alves da Silva Neto, Wanderley de Lima Pereira, Marlon Natan Baracho de Oliveira, Rodrigo Guimarães dos Santos Almeida, Raphael Raniere de Oliveira Costa

**Affiliations:** ^1^ Universidade Federal do Rio Grande do Norte, Escola Multicampi de Ciências Médicas (EMCM/UFRN) Rio Grande do Norte Brazil; ^2^ Universidade de São Paulo, Escola de Enfermagem de Ribeirão Preto São Paulo Brazil

**Keywords:** clinical debriefing, interprofessional communication, patient safety, simulation in healthcare, TALK method

## Abstract

**Aim:**

To understand healthcare professionals' perceptions regarding the application of the Debriefing TALK method in clinical practice, with emphasis on interprofessional communication and patient safety.

**Method:**

A qualitative action research study conducted with 12 healthcare professionals from secondary care in the Northeast region of Brazil. The educational intervention included dialogic lectures, communication skills training, clinical simulation, and structured debriefing sessions using the TALK method. Qualitative data were analyzed using the Collective Subject Discourse method, while quantitative data were analyzed using descriptive statistics. The study followed COREQ recommendations.

**Results:**

The TALK method functioned as a structuring device for reflective practice, promoting the reorganization of interprofessional interactions and reframing communication failures as opportunities for learning. Participants reported improvements in communication, team integration, and care planning, as well as the promotion of a psychologically safe and non‐punitive environment. Reported barriers included time constraints, workload burden, variable engagement, and limited institutional support.

**Conclusion:**

The TALK Debriefing method proved feasible and well accepted, showing potential to enhance clinical practice through structured reflection, meaningful learning, and strengthened interprofessional communication, contributing to patient safety in real‐world care contexts.

**Implications for Practice:**

The findings suggest that the TALK method may support reflective and communication‐centered practices in healthcare services, strengthening interprofessional collaboration and patient safety culture.

**Impact:**

This study advances knowledge on structured debriefing in real clinical settings, highlighting the TALK method as a pedagogical and organizational strategy capable of transforming communication failures into opportunities for collective learning and practice improvement.

**Reporting Method:**

Reported according to COREQ.

**Patient or Public Contribution:** No patient or public contribution was involved in this study.

## SUMMARY

What does this paper contribute to the wider global clinical community?

This study provides qualitative insights into healthcare professionals' experiences with structured clinical debriefing (TALK) in real‐world, high‐demand clinical settings, highlighting its role in improving communication, team integration, and reflective practice.

Findings show that systemic pressures, time constraints, variable engagement, and limited organizational support can compromise implementation, indicating that debriefing effectiveness is context‐dependent rather than inherent to the method.

Results underscore the need for integrating structured debriefing into routine care through leadership support, psychologically safe environments, and sustained educational strategies to enhance patient safety, team performance, and workforce well‐being.

## 1 Introduction

Patient safety is a central pillar of healthcare quality and remains a global priority for reducing adverse events, improving clinical outcomes, and strengthening health systems [1]. In Brazil, this movement was consolidated with the creation of the National Patient Safety Program (PNSP), established by Ordinance No. 529/2013, which sets guidelines focused on risk management, continuing education, and strengthening a safety culture (Brazil, 2013).

Among the determinants of safe care practices, effective communication and interprofessional collaboration stand out. Integration among different professional categories fosters knowledge sharing, co‐responsibility for care, and shared decision‐making, reducing failures associated with fragmented care [2, 3]. Evidence indicates that interprofessional educational interventions contribute to improving team performance and patient safety [4].

In this context, Simulation‐Based Education (SBE) has been consolidated as a robust pedagogical strategy for developing both technical and non‐technical competencies, particularly communication, leadership, teamwork, and clinical reasoning [5]. Among its components, debriefing is recognized as a central element of the learning process, as it promotes critical reflection, structured feedback, and the consolidation of learning from experience [6].

Among the debriefing models described in the literature, the TALK method (Target, Analyze, Learning points, Key actions) stands out, developed to support brief, structured, and solution‐oriented clinical discussions. Recent studies, particularly in the Latin American context, demonstrate its feasibility, multiprofessional applicability, and potential to enhance communication and teamwork [7].

However, despite the growing recognition of debriefing as an essential strategy in healthcare education, important gaps remain in the literature regarding the application of structured models, such as TALK, in real‐world interprofessional clinical practice. Most evidence is concentrated in simulated or academic environments, with limited exploration of its implementation in healthcare services and its effects on teams' daily practice [6, 8]. Furthermore, there is a scarcity of qualitative studies investigating how professionals perceive the incorporation of this method into their routine care, especially in secondary care settings and regions outside major urban centers.

Thus, the research problem guiding this study is to understand how training in the use of the TALK method may contribute to interprofessional clinical practice, particularly regarding communication and patient safety in real‐world care contexts.

Accordingly, this study aimed to train healthcare professionals in the application of the TALK method and to analyze their perceptions of its contribution to clinical practice, with emphasis on interprofessional communication and patient safety. This study contributes by advancing understanding of the operationalization of structured debriefing in real clinical settings, expanding the scope of the TALK method beyond simulated environments.

## 2 Method

### 2.1 Study Design

This is a qualitative evaluative study of educational intervention. The conduct and reporting of the study followed the recommendations of COREQ (Consolidated Criteria for Reporting Qualitative Research), ensuring methodological rigor, transparency, and completeness in the description of procedures [9].

The research team consisted of a principal investigator (a nurse with a PhD in Nursing, experienced in clinical simulation and qualitative research) and a master's student (a nurse with experience in simulation and clinical practice). There was no prior hierarchical relationship with participants. A reflexive stance was adopted throughout all stages of the study [9].

### 2.2 Setting and Participants

The study was conducted at Escola Multicampi de Ciências Médicas, Universidade Federal do Rio Grande do Norte, Brazil. Participants were healthcare professionals working in secondary care services, including physicians, nurses, nursing technicians, physiotherapists, and social workers.

A non‐probabilistic convenience sample was used, comprising 12 participants who met the eligibility criteria: professional practice in the region, full participation in the intervention, and voluntary consent. Professionals who did not complete all stages or data collection instruments were excluded.

### 2.3 Procedures

The study was carried out in four sequential stages: (1) participant recruitment, following institutional approval and dissemination of a digital invitation; (2) planning of the educational intervention; (3) implementation of the training intervention; and (4) data analysis.

### 2.4 Educational Intervention

The intervention consisted of a short training course conducted in two cohorts (November 11 and 12, 2025), with a total workload of four in‐person hours.

### 2.5 Intervention Structure

The intervention was organized into three components:

- Dialogic lecture (1 h): covering the fundamentals of clinical debriefing, patient safety, and interprofessional communication, with emphasis on the TALK method;
- Skills training (1 h): conducted using two educational videos developed by the research team, used as triggers for identifying communication failures, guided discussion, and training in cognitive and communication skills;
- Clinical simulation and structured debriefing (2 h): including simulated scenarios followed by structured debriefing sessions conducted immediately after each activity [5, 6].

## 3 TALK Debriefing Protocol

Debriefing sessions were conducted in a standardized manner, following the four stages of the TALK method:

- T—Transparency: establishment of a psychologically safe environment and alignment of expectations;
- A—Analysis: discussion of actions performed, clinical decisions, and performance;
- L—Learning: construction of meaning and integration of theory and practice;
- K—Key actions: definition of strategies applicable to professional practice.

### 3.1 Facilitators and Standardization

The intervention was delivered by the research team (principal investigator, master's student, and collaborators). Facilitators had prior experience in clinical simulation and underwent preparatory alignment, including review of the TALK method; standardization of facilitation strategies; and rehearsal of session delivery. This process ensured consistency and fidelity of the intervention [5].

### 3.2 Data Collection

Three instruments were used:

- Sociodemographic questionnaire (sex, age, profession, years of experience, prior training in patient safety);
- Structured course evaluation instrument, adapted from Diaz‐Navarro et al. [7], using a five‐point Likert scale (content clarity, instructor performance, applicability, and recommendation);
- Open‐ended questionnaire, addressing perceptions, strengths, weaknesses, and impacts of the TALK method on professional practice.

### 3.3 Data Analysis

Qualitative data were analyzed using the Collective Subject Discourse (CSD) method, according to Lefèvre and Lefèvre [10]. The analytical process included: identification of key expressions; definition of central ideas; identification of anchoring elements; organization into analytical matrices; and construction of synthesis discourses. Data collection was concluded upon reaching theoretical sufficiency.

Additionally, a qualitative interpretative matrix was developed to visually synthesize the study findings. This matrix was constructed based on the frequency of Collective Subject Discourses (CSD), the analytical density of key expressions, and the central ideas identified within thematic categories.

For each analytical domain, interpretative intensity levels were assigned (strong/predominant, emerging/moderate, punctual or divergent), considering the recurrence of discourses, internal consistency of statements, and the presence of tensions or contradictions among participants.

It should be noted that this matrix does not have a statistical nature but rather serves as an interpretative synthesis tool for qualitative data, aimed at facilitating an integrated visualization of findings and the relationships between strengths and barriers in the implementation of the method.

### 3.4 Ethical Aspects

The study was approved by the Research Ethics Committee (approval no. 7.904.383), in accordance with Resolution No. 466/2012 of the Brazilian National Health Council (Brazil, 2012). All participants provided written informed consent.

### 3.5 Use of Artificial Intelligence

An artificial intelligence tool (ChatGPT) was used exclusively to support the development of visual representations of the results. The theoretical conception, interpretation, and final validation were carried out entirely by the authors.

## 4 Results and Discussion

### 4.1 Participant Characteristics

The sample was predominantly composed of nursing professionals (50%) and female participants (83.3%), with a mean age of 33.8 years, characterizing a relatively young group at an intermediate stage of their careers. The average years since graduation (6.3 years) and time working in the sector (5.1 years) suggest a team with consolidated practical experience yet still undergoing professional development.

Heterogeneity was observed regarding prior training in patient safety (50%), indicating unequal exposure to formal content in this area. Nevertheless, the high frequency of reported communication failures (75%) was consistent across participants, suggesting that such events are not limited to specific levels of experience or training.

This finding reinforces that gaps in clinical communication may persist even among professionals with established trajectories, pointing to limitations in traditional educational approaches and the need for more experientially oriented training strategies.

Comparative analysis among participants showed that reports of communication failures were frequent regardless of prior patient safety training, indicating that exposure to formal content does not necessarily translate into effective changes in communicative practice.

Additionally, professionals with longer experience in the sector did not show an apparent reduction in the occurrence of these events, suggesting that experience alone may not be sufficient to mitigate communication failures in complex care settings. Considering the predominance of nursing professionals in the sample, it is possible that these individuals demonstrate greater sensitivity in identifying such failures, given their central role in care coordination and interprofessional communication [2].

### 4.2 Interprofessional Communication and Team Integration

Participants reported significant improvements in communication, dialogue, and team integration following the training, as evidenced in CSD 1:

“The method facilitates reflection and the correction of communication failures, strengthening dialogue and team integration. It promotes inclusion, support, and clarity among all, allowing solutions to be constructed collectively. I perceive that communication improves significantly, directly contributing to better team performance.”

This finding suggests that the TALK method functioned as a structuring device for interprofessional dialogue, fostering collective reflection, active listening, and the shared construction of solutions. This result aligns with recent evidence demonstrating that structured debriefing strengthens interprofessional communication, reduces hierarchical barriers, and promotes collaborative culture in healthcare teams [4, 7, 8].

However, this positive perception was not homogeneous. Some participants reported difficulties related to team participation and engagement:

“Not everyone feels comfortable speaking, and the lack of team engagement can compromise the entire process.”

Additionally, concerns emerged that the reflective process might be perceived as criticism:

“Depending on how the method is conducted, some people may interpret it as criticism rather than reflection.”

These findings reveal an important tension between the structuring potential of the method and the relational conditions of the work environment. In complex care settings, structured reflective conversations contribute to transforming individual perspectives into shared clinical reasoning; however, their effectiveness depends on the presence of psychological safety and openness to dialogue—essential conditions for professionals to feel comfortable sharing perceptions, questioning practices, and engaging in collective learning [11, 12].

### 4.3 Structured Reflection and Care Planning

Another key finding was the perception of greater organization and structure in clinical discussions, as illustrated in CSD 2:

“The method makes it possible to structure conversations and evaluations that previously occurred informally, offering an organized approach with clear objectives and a focus on solutions.”

Participants highlighted that the method provided greater clarity of objectives, focus on discussions, and improved direction of clinical actions. This result is particularly relevant in the Key Actions stage of the TALK method, in which reflection is intentionally translated into concrete care planning.

The literature supports this interpretation, demonstrating that structured debriefing models promote collaborative planning, improved team efficiency, and greater clinical problem‐solving capacity [6, 7]. However, limitations related to representativeness were also identified:

“The simulation did not include all those involved in real‐life situations, such as management and support staff.”

This finding suggests that, although the method organizes clinical reasoning, its effectiveness depends on the inclusion of the various stakeholders involved in care. Indeed, the quality of clinical decision‐making is directly related to the integration of professional and organizational perspectives. In this sense, structured reflective approaches tend to achieve greater impact when embedded in interprofessional practices and organizational arrangements that promote broader participation and shared responsibility for care [3, 13].

### 4.4 Learning From Errors and Safety Culture

Participants indicated that the method facilitated learning from clinical events and the prevention of future failures, as illustrated in CSD 3:

“The method allows clarification of what happened, generates learning, and helps find solutions to prevent similar situations from occurring again.”

This result aligns directly with contemporary patient safety frameworks, particularly those centered on learning systems and non‐punitive cultures. Recent studies on the implementation of TALK demonstrate significant gains in collective learning, action planning, and strengthening of safety culture [4, 7]. However, important nuances emerged. Some participants did not identify weaknesses or were unable to evaluate the method:

“I did not identify any weaknesses.”

“I have no opinion, as I have not applied the method in practice.”

These statements suggest heterogeneity in the appropriation of the strategy, potentially reflecting both high acceptance and limitations in practical experience or initial critical capacity. This finding indicates that the incorporation of reflective methods oriented toward patient safety does not occur immediately but requires progressive processes of engagement, development of reflective competencies, and consolidation of an organizational culture that values error as a learning opportunity. Thus, the effectiveness of such strategies is intrinsically related to the maturity of the safety culture and the institutional capacity to sustain continuous learning environments [14, 15, 16].

### 4.5 Professional Development and Quality of Care

Participants reported that the method contributed to active learning, increased confidence in practice, and professional development:

“The method promotes my active learning and increases my confidence in practice.”

This finding is consistent with the Simulation‐Based Education literature, which recognizes debriefing as a central stage for consolidating clinical reasoning, critical thinking, and professional self‐confidence [5, 6]. Additionally, participants perceived a direct impact on the quality of care:

“The method provides practical and objective guidance that helps the entire team apply techniques to optimize and improve service quality.”

However, the need to expand simulation activities points to limitations in consolidating these gains:

“More clinical simulations would be necessary for the method to have greater applicability.”

This result reinforces that the impact of the method depends on continuity, deliberate practice, and integration with continuing education strategies. The development of clinical competencies is not sustained through isolated interventions but through longitudinal processes of experiential learning and structured feedback. Therefore, the consolidation of observed gains is directly related to the frequency of exposure to simulated scenarios, the quality of debriefing, and the integration of these strategies into institutional training programs [5].

### 4.6 Barriers to Implementation

Despite the overall positive evaluation, relevant operational and organizational barriers were identified:

“Team availability and time itself are major challenges.”

“Dependence on other professionals, who are usually very busy, makes the process difficult to carry out.”

Relational aspects also emerged as barriers:

“Not everyone feels comfortable speaking.”

These barriers are widely described in the literature as central challenges to the sustainable implementation of clinical debriefing programs [4, 8].

On the other hand, some participants did not identify any weaknesses:

“I did not find any.”

This divergence highlights that perceptions of the method are influenced by contextual factors, individual experiences, and organizational culture. The interpretative synthesis of the findings allowed the TALK method to be understood as a structuring device for interprofessional clinical practice, promoting improvements in communication, care planning, and learning from errors. However, the results also reveal important tensions between formal knowledge and clinical practice, between the transformative potential of the method and organizational barriers, between openness to dialogue and fear of exposure, and between initial enthusiasm and implementation limitations.

These contradictions indicate that the effectiveness of the method depends on the articulation between pedagogical strategy, institutional culture, and working conditions. Educational interventions embedded in complex healthcare systems do not produce linear effects but are mediated by organizational dynamics, power relations, and contextual characteristics. In this sense, the method emerges not only as an educational technique but as a sociotechnical device capable of reorganizing collective practices by transforming critical events into opportunities for shared learning and strengthening interprofessional dynamics in complex environments (May et al., 2009).

To provide an integrative visualization of the qualitative findings, an interpretative heatmap was developed based on the frequency and analytical density of the Collective Subject Discourses (CSD), as well as the tensions identified across categories (Figure 1).

**Figure 1:**
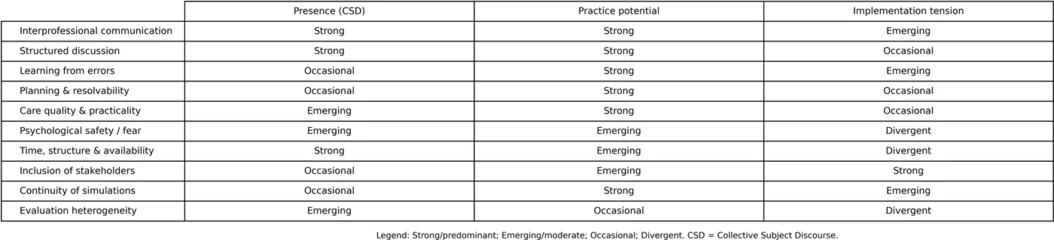
Interpretative qualitative heatmap of perceptions about the TALK method. Legend: Strong/predominant; Emerging/moderate; Occasional; Divergent. CSD = Collective Subject Discourse.

In this sense, graphical representation not only synthesizes the findings but also enables the identification of patterns of convergence and tension that would be difficult to apprehend in isolation, contributing to a more systemic understanding of the method's implementation. Furthermore, the heatmap provides guidance for implementation strategies by highlighting critical points that require organizational interventions, such as strengthening psychological safety and improving working conditions [17].

This study shifts structured debriefing from the domain of simulation to the real context of clinical practice, demonstrating that it can function as a sociotechnical device for reorganizing interprofessional work and in‐service learning.

## 5 Conclusion

The results of this study indicate that training healthcare professionals in the application of the Debriefing TALK method is feasible, well accepted, and has the potential to enhance clinical practice. The intervention contributed to improvements in interprofessional communication, team integration, and care planning—core elements for patient safety.

The qualitative analysis demonstrated that the method facilitates the structuring of clinical discussions and critical reflection on practice, particularly by enabling learning from care‐related events and the collective construction of solutions. Its role in promoting a psychologically safe environment is also noteworthy, one that prioritizes process analysis over individual blame, aligning with international recommendations for strengthening a culture of safety.

Despite the perceived benefits, barriers related to workload burden, time constraints, team engagement, and institutional support indicate that the sustainability of the strategy depends on its integration into organizational processes, with support from management and local leadership.

As a scientific contribution, this study advances knowledge by demonstrating, in a real‐world interprofessional practice context, the applicability of Debriefing TALK as a structured strategy for developing non‐technical skills, particularly communication, teamwork, and shared decision‐making. Additionally, it expands the national literature by exploring its implementation in a secondary care setting, which remains underexplored, reinforcing the role of debriefing beyond simulated environments.

Future studies are recommended to investigate the long‐term effects of the method, including care outcomes and patient safety indicators, as well as implementation strategies that support its sustainable integration across different institutional contexts.

## Author Contributions

**Jessica Santos Oliveira Targino:** conceptualization, methodology, investigation, data curation, formal analysis, writing – original draft, writing – review and editing. **Antonio Alves da Silva Neto:** investigation, data curation, writing – review and editing. **Wanderley de Lima Pereira:** investigation, data curation, writing – review and editing. **Rodrigo Guimaraes dos Santos Almeida:** writing – review and editing. **Raphael Raniere de Oliveira Costa:** conceptualization, methodology, supervision, formal analysis, writing – original draft, writing – review and editing, project administration.

## Ethics Statement

The study was approved by the Research Ethics Committee (approval no. 7,004,383), in accordance with Resolution No. 466/2012 of the Brazilian National Health Council (Brazil, 2012). All participants provided written informed consent.

## Conflicts of Interest

The authors declare no conflicts of interest.

## Data Availability

The data supporting the conclusions of this study are available from the corresponding author upon reasonable request.
